# A multidisciplinary care pathway improves quality of life and reduces pain in patients with fibrous dysplasia/McCune-Albright syndrome: a multicenter prospective observational study

**DOI:** 10.1186/s13023-022-02588-z

**Published:** 2022-12-17

**Authors:** Maartje E. Meier, Marlous Hagelstein-Rotman, Annenienke C. van de Ven, Ingrid C. M. Van der Geest, Olav Donker, Sarina E. C. Pichardo, Petra C. E. Hissink Muller, Stijn W. van der Meeren, Desirée M. J. Dorleijn, Elizabeth M. Winter, Michiel A. J. van de Sande, Natasha M. Appelman-Dijkstra

**Affiliations:** 1 Center for Bone Quality, Department of Orthopaedic Surgery, Leiden University Medical Center, Leiden, The Netherlands; 2 Division of Endocrinology, Department of Internal Medicine, Center for Bone Quality, Leiden University Medical Center, Leiden, The Netherlands; 3 Department of Endocrinology, Radboud University Medical Center, Nijmegen, The Netherlands; 4 Department of Orthopaedic Surgery, Radboud University Medical Center, Nijmegen, The Netherlands; 5 Department of Oral and Maxillofacial Surgery, Leiden University Medical Center, Leiden, The Netherlands; 6 Department of Paediatric Rheumatology, Willem Alexander Children’s Hospital, Leiden University Medical Center, Leiden, The Netherlands; 7 Department of Ophthalmology, Leiden University Medical Center, Leiden, The Netherlands

**Keywords:** Fibrous dysplasia, McCune-Albright syndrome, Rare bone disease, Care pathway, Multidisciplinary care, Tertiary referral hospital, Quality of life, Pain, Patient reported outcome measure

## Abstract

**Background:**

Fibrous dysplasia/McCune-Albright syndrome (FD/MAS) may cause pain, impaired ambulation and decreased quality of life (QoL). International guidelines advocate management of FD/MAS in a tertiary multidisciplinary care pathway, but no longitudinal data are available to support this recommendation. This multicenter prospective observational study aimed to evaluate effects of 1 year of treatment in the FD/MAS care pathway in 2 tertiary clinics on QoL and pain, assessed by change in Short Form 36 and Brief Pain Inventory between baseline and follow-up. Patients completing baseline questionnaires < 1 year after intake were classified as new referrals, others as under chronic care.

**Results:**

92 patients were included, 61 females (66%). 22 patients (24%) had monostotic disease, 16 (17%) isolated craniofacial FD, 27 (40%) polyostotic FD and 17 (19%) MAS. 26 were new referrals (28%) and 66 chronic patients (72%). Median age at baseline was 47 years (Q1–Q3 36–56). Skeletal burden correlated with baseline Physical Function (r_s_ = − 0.281, *p* = 0.007). QoL was in all domains lower compared to the general population. New referrals reported clinically important differences (CID) over time in domains Physical Function (mean 67 ± SD24 to 74 ± 21, effect size (ES) 0.31, *p* = 0.020), Role Physical (39 ± 41 to 53 ± 43, ES 0.35, *p* = 0.066), Social Functioning (64 ± 24 to 76 ± 23, ES 0.49, *p* = 0.054), and Health Change (39 ± 19 to 53 ± 24, ES 0.76, *p* = 0.016), chronic patients in Physical Function (52 ± 46 to 66 ± 43, ES 0.31, *p* = 0.023) and Emotional Wellbeing (54 ± 27 to 70 ± 15, ES 0.59, *p* < 0.001). New referrals reported a CID of 1 point in maximum pain, average pain and pain interference, chronic patients reported stable scores. Change in pain interference and Role Physical were correlated (r_s_ = − 0.472, *p* < 0.001). Patients with limited disease extent improved more than patients with severe disease. Patients receiving FD-related therapy had lower baseline scores than patients not receiving therapy and reported improvements in QoL after 1 year. Yet also patients without FD-related therapy improved in Physical Function.

**Conclusions:**

All FD-subtypes may induce pain and reduced QoL. A multidisciplinary care pathway for FD/MAS may improve pain and QoL, mainly in new referrals without MAS comorbidities with low baseline scores. Therefore, we recommend referral of patients with all subtypes of FD/MAS to specialized academic centers.

**Supplementary Information:**

The online version contains supplementary material available at 10.1186/s13023-022-02588-z.

## Background

Fibrous dysplasia (FD), is a rare, congenital bone disease, where fibrous skeletal lesions develop due to postzygotic mutations in the *GNAS* gene and subsequent aberrant bone turnover [1, 2]. The genetic mosaicism leads to a variable range of skeletal involvement and to a heterogeneous presentation, ranging from a single affected bone (monostotic FD) to severe disease with multiple bones involved (polyostotic FD) [3]. Because of the asymptomatic subset of patients, the prevalence remains unknown. In symptomatic patients pain and impaired mobility are common, as well as susceptibility to fractures or deformity. Additional extraskeletal manifestations of the GNAS mutation may be present in the McCune-Albright Syndrome (MAS), including endocrinopathies and skin hyperpigmentation [3, 4]. Recently, management guidelines have been established by the FD/MAS international consortium to address the diagnostic and therapeutic challenges emerging from the rarity of the disease, complex multiorgan involvement and heterogeneous phenotype [5]. This guideline provides a care pathway for diagnosis, staging, monitoring and treatment to alleviate symptoms. The provided treatment protocols consist of general measures, pharmacological therapy and surgical interventions, and are based on expert opinion and published studies [5, 6]. Most of these studies address objective outcome measures such as bone turnover markers after medical therapy [7–10], or radiologic outcomes or revisions after surgery [11–13]. However, no studies have evaluated the effect of treatment on patient reported outcome measures (PROMs) or the longitudinal follow-up of PROMs in FD/MAS in general. Yet, this topic cannot be neglected since patients with FD/MAS report impairments in several domains of quality of life (QoL) compared to the general population [14] and more negative illness perceptions [15, 16]. These psychosocial consequences correlate with disease severity [14–16], but may also be influenced by inadequate information, misdiagnosis, diagnostic delay or ineffective treatment, problems which arise frequently due to the rarity and heterogeneity of FD/MAS [5]. In addition, the follow-up of PROMs is important to support the recommendation to manage FD/MAS in a specialized center. The Leiden University Medical Center (LUMC) and Radboud University Medical Center (RUMC) are both tertiary referral centers for FD/MAS and have implemented the care pathway as proposed in the guidelines, to tackle all physical and psychosocial aspects of FD/MAS, to minimize diagnostic and therapeutic delay, and to improve (patient reported) outcomes. Patients are provided with information and counselling and the diagnostic and therapeutic strategy advocated in the guidelines are coordinated in a multidisciplinary setting. Care pathways have been successful for other diseases and have shown to provide enhanced adherence to guidelines and documentation of care [17–19], reduced variability in care [20, 21], improved clinical outcomes [18, 20–23] and better teamwork [24]. For FD/MAS the value of a care pathway has not yet been established. For this reason, we aimed to evaluate the effect of the LUMC/RUMC multidisciplinary pathway on QoL and pain in patients with FD/MAS, as we expected improvements compared to usual care. Secondary aims were to compare QoL with the general population, to assess differences between FD subtypes, to compare patients with and without FD-related therapy during follow-up and evaluate illness perceptions.

## Methods

### Population

This study was conducted between January 2018 and August 2021 and was performed within the PROFID-study, an ongoing multicenter prospective observational cohort study into patients with FD/MAS. The PROFID started in 2017 and is conducted on the outpatient clinics of Endocrinology and Orthopaedic Surgery of the LUMC and RUMC. Inclusion criteria are a certain diagnosis of FD/MAS (monostotic FD, polyostotic FD or MAS) and at least 1 visit at one of the outpatients clinics. In the current cohort we only presented results of the adult population. Written informed consent is required for inclusion. The study was approved by the Medical Ethics Committee of both centers (protocol number P17.136). Several patients have been included in previous studies [14–16, 25, 26], but data were not reused and generated originally for this study.

### Research materials

Patients were asked to complete questionnaires upon inclusion in the study and again after 1 year of care in the FD/MAS care pathway. If follow-up visits were not deemed necessary or were planned further ahead than 1 year, questionnaires were send regardless of outpatient clinic visits, 1 year after completion of the baseline questionnaire. The questionnaire set included the Short Form 36 (SF-36) [27] and EuroQoL-5D-3L (EQ-5D) [28] for quality of life, the Brief Pain Inventory (BPI) [29] and the Illness Perceptions Questionnaire-Revised (IPQ-R) [30]. The SF-36 includes physical health domains (limitations in physical activities due to health problems (‘Physical Function’), bodily pain (‘Pain’) and limitations in usual role activities due to physical health problems (‘Role Physical’)); mental health domains (vitality (‘Energy/fatigue’), general mental health (‘Emotional wellbeing’), limitations in usual role activities due to emotional problems (‘Role Emotional’), limitations in social activities due to emotional or physical problems (‘Social Functioning’)); and general health domains (self-reported general health (‘General health’) and health change compared to 1 year ago). The range is 0–100 on each domain and higher scores reflect better QoL. This is similar for the EQ-5D health state, where patients are asked to rate their health state at that moment on a visual analogue scale, 0 reflecting the worst possible health state and 100 the best state. In addition, the EQ-5D comprises 5 subdomains on Mobility, Self-care, Usual Activities, Pain/Discomfort and Anxiety/Depression. Self-perceived problems can be scored on each domain on 3 levels: 1) no problems, 2) some problems, or 3) extreme problems. A summary score can be calculated with a publicly available formula that attaches weights to each level in each dimension. For this study the summary score and health state were used. The BPI is divided in a part on pain severity, where patients score their worst pain, least pain, pain on average and pain right now, and on interference of pain with functioning (e.g. general activity, mood, work). The range is 0–10, higher scores reflect more pain or more pain interference. We considered the items maximum pain, average pain and pain interference with general activity most relevant for our research and excluded other domains. For the IPQ-R the range is 6–30 for Timeline Acute/Chronic, Consequences, Personal Control and Emotional Representation, 4–20 for timeline cyclical, 5–25 for treatment control and illness coherence. Higher scores for Personal/Treatment Control and Illness Coherence indicate positive beliefs about controllability and understanding of the disease, whereas higher scores on Timeline Acute/Chronic/Cyclical, on Consequences and on Emotional Response represent strong beliefs on the acute/chronic/cyclical nature and on negative consequences and emotions caused by the disease. Baseline characteristics including FD subtype, FD-related comorbidity, skeletal burden score (SBS) [31] and treatment history were retrieved from electronic health records.

### Research methods

For this study, patients were selected who completed at least one questionnaire at both time points. The primary endpoint was the change in SF-36 and BPI scores during the 1 year of follow-up. This endpoint is assessed in 2 groups: patients were regarded as new referrals if the baseline questionnaire was completed within 1 year after intake in the hospital and all other patients were classified as under chronic care. This distinction was made because in the newly referred group, the baseline scores for QoL and pain can be used as proxy for the level of QoL and pain at the end of the care trajectory elsewhere. In this way the temporal change during follow-up was regarded to reflect the added value of the FD/MAS care pathway in comparison to usual care. Change in QoL and pain in chronic patients was assessed to observe baseline differences between groups and longitudinal change in this subgroup. For the SF-36 the clinically important difference (CID) was expressed as effect size (ES) and a threshold of 0.20 was used [32], for the BPI the CID was considered 1 point reduction [33]. Secondary endpoints were differences in SF-36 scores between the FD/MAS cohort and the general population, temporal change in EQ-5D scores, baseline and temporal differences in SF-36 and pain scores between FD subtypes (monostotic non-craniofacial FD (MFD), isolated craniofacial FD (CFD), PFD and MAS) and between treatment groups during follow-up (FD-related therapy during follow-up or no therapy), and lastly differences in IPQ-R scores between referral groups and over time.

### Diagnostic and therapeutic protocol of the FD/MAS care pathway

Patients are referred to the LUMC or RUMC by general practitioners, non-academic hospitals or academic hospitals. The diagnosis, often established in the referring center, is made on clinical, biochemical, radiographical or pathological parameters. During the 1 year of follow-up in this study, patients were staged and treated in the FD/MAS care pathway according to the guidelines [5]. The FD/MAS team consists of endocrinologists and orthopaedic surgeons who are experienced and specialized in FD/MAS. Close multidisciplinary collaboration allows for comprehensive screening of the patient; for a rapid management plan; and for thorough provision of background information with counselling and reassurance. Patients are screened for complaints, skeletal complications or additional endocrinopathies. Bone-related laboratory testing is performed upon intake, during active surveillance, and before and after treatment. Imaging of the lesions is conducted and skeletal scintigraphy or ^18^F-NaF PET/CT [34] to determine disease extent. If indicated, a patient-tailored assessment of vision, hearing, endocrine function or other extraskeletal morbidity is conducted with consultation of relevant specialists including ophthalmologists, oral- and maxillofacial surgeons, neurosurgeons and rehabilitation physicians. Asymptomatic patients remain under surveillance unless wishing to return to the referring center. Symptomatic patients may be treated with physical therapy or rehabilitation; with medical therapy such as analgesics, supplements of phosphate/vitamin D/calcium, bisphosphates or as a last resort denosumab; or with surgery for mechanical pain, (impending) fracture, deformity or nerve compression. A detailed description is provided in the guidelines [5].

### Statistical analysis

Categorical data are presented as number (percentage), numerical data as mean (± SD) or median (quartile 1-quartile 3). SF-36 scores were compared with the general Dutch population by one-sample t-tests. Numerical data were in case of normality compared between time points with paired t-tests, between 2 groups with independent t-tests, and between the 4 FD subtypes by one-way ANOVA, whereas in absence of normality Wilcoxon Signed rank, Mann–Whitney U and Kruskal Wallis tests were used respectively. Categorical data were compared with Chi Square tests. Analyses concerning pain were conducted in patients with pain (baseline maximum or average pain score ≥ 0) and the subgroup of patients with moderate to severe pain (score ≥ 4/10), because the pain severity in the latter group is an indication for analgesic therapy. Subgroups for FD-related treatment during follow-up were compared on the presence or absence of antiresorptive or surgical treatment during the 1 year in the care pathway. The correlation between SBS and SF-36 domain Physical Function at baseline was assessed by Spearman’s rank-order correlation, as well as the correlation between temporal change in pain interference and in Role Physical. Correction for multiple testing was not performed because of the exploratory nature of the study, because the CID was deemed more important, and to omit the misuse of the *p*-value as dichotomizing instrument [35, 36]. Missing data were excluded and analysed by comparing characteristics of complete cases to patients with no questionnaires or questionnaires completed a merely 1 time point.

## Results

### Patient characteristics

92 consecutive patients were included in this study, 26 new referrals (28.3%) and 66 patients under chronic care (72.7%) (Table 1). 85 patients (92%) completed all questionnaires and 7 patients (8%) filled in questionnaires at both time points but incompletely. Median age at baseline was 46.5 (Q1-Q3 36–56) and comparable between referral groups. The total cohort consisted of 61 females (66.3%). 22 patients (24%) were diagnosed with MFD, 16 (17%) with CFD), 27 (40%) with PFD and 17 (19%) with MAS. Patients in the chronic group were more frequently affected with PFD or MAS. Median SBS was 3.2 (1.2–16.9) and comparable between groups. SBS correlated with SF-36 domain Physical Function at baseline (Spearman’s rho =0.281, *p* = 0.007). Most patients were included in the LUMC (n = 77, 83.7%) and were referred from a non-academic hospital (n = 42, 46%) or from another academic hospital (n = 23, 25%). Mean follow-up (time between survey completion) was 1.2 (± SD 0.3) years. The majority of patients had received FD-related treatment prior to inclusion in this study, specifically bisphosphonates (n = 68, 74%), denosumab (n = 20, 22%) or surgery (n = 40, 44%). Half of the patients had received FD-related treatment during follow-up: 35 patients (38%) received bisphosphonates, 16 (17%) denosumab and 5 (5%) surgery, whereas 42 patients (46%) did not receive FD-related therapy.

**Table 1:** Baseline characteristics

	New (n = 26)	Chronic (n = 66)	Total (n = 92)
Age at baseline years, median (Q1-Q3)	47.5 (28–59)	46 (37–55)	46.5 (36–56)
Sex, female n (%)	11 (42%)	50 (76%)	61 (66.3%)
Type of FD ^a^ n (%)			
MFD (non-CFD)	9 (34.6%)	13 (19.7%)	22 (23.9%)
Isolated CFD	7 (26.9%)	9 (13.6%)	16 (17.4%)
PFD	8 (30.8%)	29 (43.9%)	37 (40.2%)
MAS	2 (7.7%)	15 (22.7%)	17 (18.5%)
Skeletal Burden Score ^b^ median (Q1-Q3)	3.2 (0.5–15.8)	4.8 (1.2–17.1)	3.2 (1.2–16.9)
Hospital n (%)			
LUMC ^c^	25 (96.2%)	52 (78.8%)	77 (83.7%)
RUMC ^d^	1 (3.8%)	14 (21.2%)	15 (16.3%)
Referring physician n (%)			
General practitioner	4 (15.4%)	10 (15.2%)	14 (15.2%)
Non-academic hospital	14 (53.8%)	28 (42.4%)	42 (45.7%)
Academic hospital	7 (26.9%)	16 (24.2%)	23 (25%)
Unknown	1 (3.8%)	12 (18.2%)	13 (14.1%)
Time between surveys years, mean (± SD)	1.3 (0.4)	1.2 (0.3)	1.2 (0.3)
Treatment history prior to inclusion n (%)			
Bisphosphonates	16 (61.5%)	52 (78.8%)	68 (73.9%)
Denosumab	1 (3.8%)	19 (28.8%)	20 (21.7%)
Surgery	7 (26.9%)	33 (50%)	40 (43.5%)
None of the above	9 (34.6%)	8 (12.1%)	17 (18.5%)
Treatment during follow-up n (%)			
Bisphosphonates	15 (57.7%)	20 (30.3%)	35 (38%)
Denosumab	1 (3.8%)	15 (22.7%)	16 (17.4%)
Surgery	2 (7.7%)	3 (4.5%)	5 (5.4%)
None of the above	10 (38.5%)	a32 (48.5%)	42 (45.7)

^a^*MFD* Monostotic fibrous dysplasia, *CFD* Craniofacial FD, *PFD* Polyostotic FD, *MAS* McCune-Albright syndrome

^b^range: 0–100

^c^*LUMC* Leiden University Medical Center

^d^*RUMC* Radboud University Medical Center

### Primary outcome—temporal change in SF-36 scores in new referrals and chronic patients

**Fig. 1:**
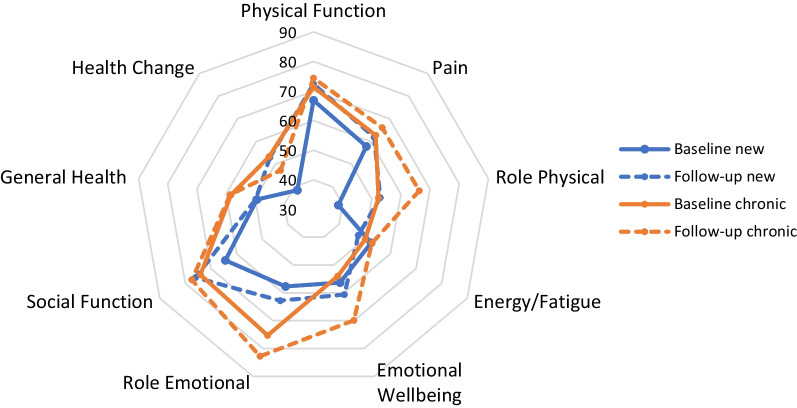
SF-36 domains at baseline and follow-up in new and chronic patients

New referrals reported clinically important improvements in Physical Function after 1 year of follow-up, from mean 67 (± SD 24) to 74 (± 21) (*p* = 0.020) (Table 2, Fig. 1). Chronic patients reported slightly higher Physical Function at baseline compared to new referrals, but no improvements during follow-up, resulting in similar scores after 1 year (Table 2, Fig. 1). Role Physical was at baseline higher in chronic patients, and in both groups a clinically relevant improvement of 14 points was observed. Domains Pain and Energy/Fatigue were in both groups similar at baseline and stable over time. Emotional Wellbeing was comparable between groups at baseline and improved more in chronic patients, from 54 (± 27) to 70 (± 15) (*p* < 0.001), compared to new referrals, 56 (± 21) to 60 (± 17) (*p* = 0.275). Social Functioning improved in new referrals from 64 (± 24) to 76 (± 23) (*p* = 0.054), was in chronic patients higher at baseline but stable over time, and was therefore comparable between groups after 1 year. Role Emotional and General Health were at baseline higher in chronic patients and in both groups stable over time. In contrast, Health Change improved in new referrals from 39 (± 19) to 53 (± 24) (*p* = 0.016), whereas chronic patients reported no change.

**Table 2:** Quality of life scores of Short Form 36*

	Baseline mean (± SD)	Follow up mean (± SD)	Difference mean (95% CI)	Effect size	*p*-value ^a^
*Physical function*					
New (n = 26)	66.9 (23.6)	74.2 (20.9)	+ 7.3 (1.2–13.4)	0.31 ^+^	*p* = 0.020
Chronic (n = 66)	71.4 (24.6)	74.4 (23.3)	+ 3.0 (− 0.61–6.5)	0.12	*p* = 0.102
Difference mean (95% CI)	4.5 (− 6.7–15.7)	0.2 (− 10.3–10.6)			
*p*-value ^c^	*p* = 0.425	*p* = 0.975			
*Pain*					
New (n = 26)	57.8 (25.9)	61.9 (23.7)	+ 4.1 (− 4.9–13.1)	0.16	*p* = 0.360
Chronic (n = 66)	62.7 (26.3)	66.1 (25.2)	+ 3.3 (− 2.4–9.1)	0.13	*p* = 0.251
Difference mean (95% CI)	5.0 (− 7.1–17.0)	4.2 (- 7.2–15.6)			
*p*-value ^c^	*p* = 0.415	*p* = 0.463			
*Role limitations due to physical problems*					
New (n = 26)	38.5 (40.8)	52.9 (43.2)	+ 14.4 (− 1–29.8)	0.35 ^+^	*p* = 0.066
Chronic (n = 66)	52.3 (45.5)	66.3 (43.1)	+ 14.0 (2.0–26.1)	0.31 ^+^	*p* = 0.023
Difference mean (95% CI)	13.8 (− 5.8–33.4)	13.4 (- 6.4–33.2)			
*p*-value ^c^	*p* = 0.163	*p* = 0.183			
*Energy/fatigue*					
New (n = 26)	52.5 (20.6)	47.5 (15.9)	− 5.0 (− 12.8–2.8)	0.24^+^	*p* = 0.199
Chronic (n = 66)	50.2 (20)	52.8 (17.8)	+ 2.6 (− 3.0–8.1)	0.13	*p* = 0.358
Difference mean (95% CI)	2.3 (− 11.5–7.0)	5.3 (- 2.7–13.3)			
*p*-value ^c^	*p* = 0.627	*p* = 0.189			
*Emotional wellbeing*					
New (n = 26)	56.3 (20.7)	60.6 (16.6)	+ 4.3 (− 3.7–12.3)	0.21^+^	*p* = 0.275
Chronic (n = 66)	54.0 (27.1)	69.9 (15.1)	+ 15.9 (9.2–22.7)	0.59^+^	*p* < 0.001
Difference mean (95% CI)	− 2.3 (− 12.8–8.2)	9.3 (2.2–16.5)			
*p*-value ^c^	*p* = 0.662	*p* = 0.011			
*Role limitations due to emotional problems*					
New (n = 26)	57.7 (42.7)	62.8 (44.5)	+ 5.1 (-− 14.2–24.4)	0.12	*p* = 0.589
Chronic (n = 66)	75.3 (36.2)	82.8 (32.7)	+ 7.6 (− 2.0–17.2)	0.21 ^+^	*p* = 0.121
Difference mean (95% CI)	17.6 (0.2–35.1)	20.0 (0.5–39.5)			
*p*-value ^c^	*p* = 0.050	*p* = 0.045			
*Social Functioning*					
New (n = 26)	64.4 (23.6)	76.0 (22.6)	+ 11.5 (− 0.3–23.3)	0.49 ^+^	*p* = 0.054
Chronic (n = 66)	74.1 (24.2)	77.5 (21.4)	+ 3.4 (− 1.2–8.0)	0.14	*p* = 0.146
Difference mean (95% CI)	9.6 (− 1.5–20.7)	1.5 (- 8.5–11.5)			
*p*-value ^c^	*p* = 0.088	*p* = 0.766			
*General Health*					
New (n = 26)	49.4 (20.9)	49.8 (17.4)	+ 0.4 (− 6.1–6.9)	0.02	*p* = 0.903
Chronic (n = 66)	58.3 (20.6)	58.6 (19.9)	+ 0.2 (− 3.0–3.5)	0.01	*p* = 0.889
Difference mean (95% CI)	8.9 (− 0.6–18.4)	8.8 (- 0.1–17.6)			
*p*-value ^c^	*p* = 0.066	*p* = 0.053			
*Health change compared to 1 year ago*					
New (n = 26)	38.5 (19.0)	52.9 (23.8)	+ 14.4 (2.9–25.9)	0.76 ^+^	*p* = 0.016
Chronic (n = 66)	48.1 (21.6)	47.3 (19.2)	− 0.76 (− 7.9–6.4)	0.03	*p* = 0.833
Difference mean (95% CI)	9.6 (0.0–19.3)	− 5.5 (− 15–3.9)			
*p*-value^c^	*p* = 0.050	*p* = 0.248			

^*^Higher scores reflect better QoL. Range: 0–100

^a^Paired samples t-test,

^b^One-sample t-test,

^c^Independent samples t-test

^+^Clinically important difference, effect size above threshold of 0.20

### Primary outcome—temporal change in BPI scores in new referrals and chronic patients

**Fig. 2:**
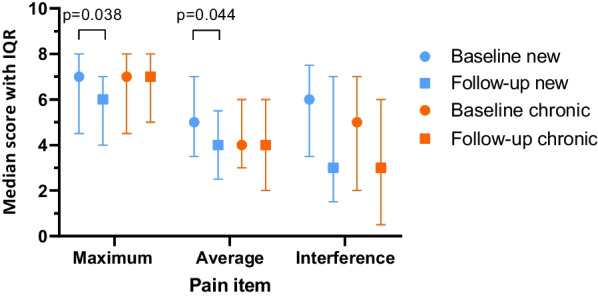
Pain scores at baseline and follow-up in new and chronic patients with moderate to severe pain

**Fig. 3:**
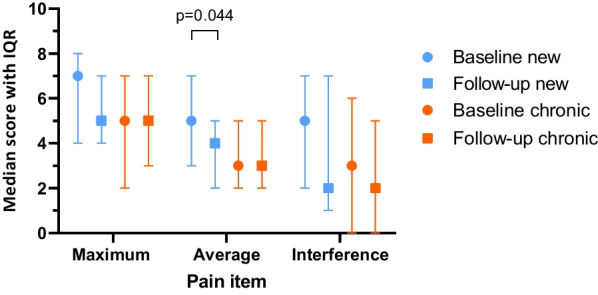
Pain scores at baseline and follow-up in new and chronic patients with pain score > 0

In the combined cohort 62 patients (67%) reported moderate to severe pain at baseline. New referrals in this subgroup reported a decreased of 1 point (Q1–Q3 0.5–3) in maximum pain (*p* = 0.038), 1 point (0–2.5) in average pain (*p* = 0.044) and 1 point (- 0.5–3) in interference of pain with general activity, which decreased from median 6 (3.5–7.5) to 3 (1.5–7) (*p* = 0.090) (Table 3, Fig. 2). In chronic patients with moderate to severe pain, scores did not change for maximum and average pain, but pain interference improved from 5 (2–7) to 3 (0.5–6) (*p* = 0.083) although median change was 0 (− 2.5–1) (Table 3, Fig. 2). The improvements in new referrals resulted in comparable scores in both groups after 1 year for average pain and pain interference. In new referrals reporting pain (score > 0), a decrease of 1 point was also observed in all 3 pain items (Table 4, Fig. 3). The largest improvement was observed in pain interference from median 5 (2–7) to 2 (1–7) (*p* = 0.099). Chronic patients had better scores at baseline and no temporal change, hence scores were similar in both groups after 1 year. In the total cohort, a moderate correlation was observed between temporal change in pain interference and in SF-36 domain Role Physical (Spearman’s rho = − 0.472, *p* < 0.001).

**Table 3:** Pain scores of Brief Pain Inventory in new and chronic patients with moderate to severe pain *

	Baseline median (Q1-Q3)	Follow up median (Q1-Q3)	Difference median (Q1-Q3)	*p*-value ^a^
*Maximum pain*				
Total (n = 62)	7 (4.75–8)	6 (4–8)	0 (− 2–1)	*p* = 0.077
New (n = 21)	7 (4.5–8)	6 (4–7)	− 1 (− 3–0.5)	*p* = 0.038
Chronic (n = 41)	7 (4.5–8)	7 (5–8)	0 (− 2–1)	*p* = 0.512
*p*-value ^b^	*p* = 0.976	*p* = 0.132		
*Average pain*				
Total (n = 62)	4.5 (3–6)	4 (2–6)	0 (− 2–1)	*p* = 0.015
New (n = 21)	5 (3.5–7)	4 (2.5–5.5)	− 1 (− 2.5–0)	*p* = 0.044
Chronic (n = 41)	4 (3–6)	4 (2–6)	0 (− 1.5–1)	*p* = 0.140
*p*-value ^b^	*p* = 0.178	*p* = 0.976		
*Interference of pain with general activity*				
Total (n = 62)	5 (2.75–7)	3 (1–6)	− 1 (− 2.25–1)	*p* = 0.016
New (n = 21)	6 (3.5–7.5)	3 (1.5–7)	− 1 (− 3–0.5)	*p* = 0.090
Chronic (n = 41)	5 (2–7)	3 (0.5–6)	0 (− 2.5–1)	*p* = 0.083
*p*-value ^b^	*p* = 0.284	*p* = 0.674		

^*^In patients with moderate to severe pain at baseline (maximum or average pain > 4). Higher scores = more pain and more interference. Range 0–10

^a^Wilcoxon signed rank test

^b^Mann Whitney U test

**Table 4:** Pain measured with Brief Pain Inventory in patients with pain score > 0 *

	Baseline median (Q1-Q3)	Follow up median (Q1-Q3)	Difference median (Q1-Q3)	*p*-value ^a^
*Maximum pain*				
Total (n = 84)	6 (3–7.75)	5 (3–7)	0 (− 2–1)	*p* = 0.656
New (n = 23)	7 (4–8)	5 (4–7)	− 1 (− 3–1)	*p* = 0.074
Chronic (n = 61)	5 (2–7)	5 (3–7)	0 (− 1–1.5)	*p* = 0.519
*p*-value ^b^	*p* = 0.078	*p* = 0.984		
*Average pain*				
Total (n = 84)	3.5 (2–5)	3 (2–5)	0 (− 1–1)	*p* = 0.174
New (n = 23)	5 (3–7)	4 (2–5)	− 1 (− 2–0)	*p* = 0.044
Chronic (n = 61)	3 (2–5)	3 (2–5)	0 (− 1–1)	*p* = 0.816
*p*-value ^b^	*p* = 0.013	*p* = 0.514		
*Interference of pain with general activity*				
Total (n = 84)	4 (1–7)	2 (0.25–6)	0 (− 2–1)	*p* = 0.080
New (n = 23)	5 (2–7)	2 (1–7)	− 1 (− 2–1)	*p* = 0.099
Chronic (n = 61)	3 (0–6)	2 (0–5)	0 (− 2–1)	*p* = 0.326
*p*-value ^b^	*p* = 0.072	*p* = 0.363		

^*^In patients with pain at baseline (maximum or average pain > 0). Higher scores = more pain and more interference. Range 0–10

^a^Wilcoxon signed rank test

^b^Mann Whitney U test

### Secondary outcome—comparison of SF-36 with general population

In the total cohort, significant and clinically relevant impairments in all domains of QoL were observed compared to the general Dutch population [37] (Additional file 1: Table A, Additional file 2: Fig. A). Differences were most outspoken in the domains Emotional Wellbeing (FD/MAS 55 ± 25, population 77 ± 17, *p* < 0.001), Energy/Fatigue (FD/MAS 51 ± 20, population 69 ± 19, *p* < 0.001), Role Physical (FD/MAS 49 ± 44, population 76 ± 36, *p* < 0.001) and General Health (FD/MAS 56 ± 21, population 71 ± 21, *p* < 0.001). After 1 year, scores improved in several domains but did not reach the level of the general population.

### Secondary outcome—temporal change in EQ-5D

In new referrals, the EQ-5D health state increased from median 68 (Q1–Q3 44–90) to 80 (59–91), *p* = 0.088. The summary score and all scores for chronic patients remained constant (data not shown).

### Secondary outcome—QoL across FD subtypes

Patients with isolated CFD reported better Physical Function (84 ± SD23) compared to patients with MFD (63 ± 31), PFD (70 ± 19) or MAS (67 ± 24) (*p* = 0.050) (Additional file 1: Table B & Additional file 3: Fig. B). Physical Function improved during follow-up in all subtypes except for MAS. Scores for the Pain subscale were comparable across subtypes and also improved least in MAS. In patients with MAS, Role Physical was least experienced at baseline and stable over time. On the contrary, all other subtypes reported improvements in Role Physical by 17–21 points during follow-up. In domain Energy/Fatigue, patients with PFD scored worst at baseline (47 ± 20) but experienced most improvement at follow-up (54 ± 26) (*p* = 0.013). In other FD subtypes scores remained stable. Emotional Wellbeing was most impaired in patients with MFD (49 ± 29) and MAS (50 ± 29) and improved in both groups to 63 (± 19) (*p* = 0.05) and to 71 (± 11) (*p* = 0.017) respectively. Also patients with PFD reported higher scores for Emotional Wellbeing at follow-up. Role Emotional was similar between subtypes and time points. Social Functioning was most impaired in MFD and CFD and improved in all subtypes except MAS. General Health did not differ between FD subtypes and no improvements were observed at follow-up. All patients except for those with MAS reported slightly better health than 1 year ago at follow-up than at baseline.

### Secondary outcome—pain across FD subtypes

In patients with moderate to severe pain, for whom analgesic therapy is indicated, scores for maximum pain were comparable across FD subtypes (Additional file 1: Table C, Additional file 4: Fig C). Average pain was least severe in patients with CFD (median 3, Q1-Q3 3–4) and most in MFD (5, 3–7) and MAS (5, 3–6), *p* = 0.165. Similarly pain interference was least experienced in CFD (4, 2–7) and most in MFD (7, 4–8). In patients reporting pain with score > 0, patients with PFD reported the highest score for maximum pain of 7 (3–7.5), versus 5 (2.5–8) in patients with MFD, 5.5 (2–7) for CFD and 5 (1–8) for MAS (Additional file 1: Table D & Additional file 5: Fig. D). Average pain scores were comparable across diagnoses, and pain interference was least experienced by patients with CFD and MAS (both median 2.5) compared to patients with MFD and PFD (median 4).

### Secondary outcome—QoL for treatment received during follow-up

In the entire cohort 50 patients (54.3%) received FD-related therapy during the 1 year of follow-up (antiresorptive therapy: n = 45, 48%, surgery: n = 5, 5%) (Additional file 1: Table E & Additional file 6: Fig. E). In patients receiving therapy, Physical Function was lower at baseline (65 ± 24) compared to patients receiving no therapy (77 ± 23, *p* = 0.041), but improved during follow-up to 71 (± 24) (*p* = 0.007). Similarly scores for Role Physical were lower in patients receiving treatment, although in this domain remarkably the greatest improvement was observed in patients not receiving therapy (55 ± 44 to 74 ± 38, *p* = 0.004, ES 0.47). Emotional Wellbeing and Social Functioning were both lower at baseline but improving during follow-up in patients with FD-related therapy.

### Secondary outcome—pain for treatment received during follow-up

Patients receiving FD-related therapy during follow-up had worse scores for maximum pain, average pain and pain interference at baseline compared to patients without therapy (Additional file 1: Table F & Additional file 7: Fig F), as expected since it was part of the treatment indication. In the therapy group the median of all scores decreased slightly during follow-up, mainly pain interference from 5 (2–7) to 3 (1–6) (*p* = 0.061), but with median difference of 0. No change was observed in patients without FD-related therapy.

### Secondary outcome—temporal change in illness perceptions

In general scores for illness perceptions were comparable over time for both new referrals and chronic patients (Additional file 1: Table G). No differences were observed between FD subtypes.

### Missing data analysis

The 92 patients with questionnaires at both time points were retrieved from a cohort of 124 patients who were included in the PROFID-study before August 1^st^ 2020 and received both questionnaires, providing a response rate of 74%. 26 of the excluded patients completed only the first questionnaire (21% of total) and 6 patients (5%) completed none after 2 reminders. Complete cases were slightly older than excluded patients, but the sex ratio and FD subtype were comparable between groups (Additional file 1: Table H). Completers did not differ from dropouts in baseline QoL or pain (Additional file 1: table H).

## Discussion

This study aimed to examine the effect of a coordinated care pathway, with information provision, counseling, and a multidisciplinary care as described in the guidelines, on QoL and pain in patients with FD/MAS. At baseline, patients with FD/MAS reported significant and clinically relevant impairments in all domains of QoL and the majority reported moderate to severe pain. After referral to our tertiary FD clinics, patients reported a clinically important difference in SF-36 domains Physical Function, Role Physical, Social Functioning and Health Change as well as a clinically relevant decrease of 1 point in maximum pain, average pain and pain interference. The baseline scores in our study reflect the level of QoL and pain accomplished during usual care in the referring secondary care facility, as the questionnaires were completed shortly after referral to our hospital prior to treatment, and therefore these data suggest that treatment in our tertiary FD care pathway for FD/MAS may benefit QoL and pain compared to usual care in other hospitals. Remarkably the SF-36 domain Pain did not reflect the improvements observed in the BPI. This SF-36 domain consists of 2 questions on pain intensity in 6 response categories and pain interference in 5. This scale may not have been sufficiently responsive to change to capture the 1-point change demonstrated by the BPI. Yet the improvements in the domains interference of pain with general activity and in Role Physical appeared to be larger than in pain and Physical Function, which implies that the FD/MAS care pathway mainly improves negative consequences of pain and impaired mobility rather than the item itself. The moderate correlation between change in pain inference and in Role Physical domain underlines this observation. Another striking finding is the discrepancy between stable scores for General Health and clinically relevant increases in Health Change. The General Health domain consists of items on self-perceived health status, tendency to become ill compared to others and expectations on future health, whereas the Health Change domain consists of 1 item on health status compared to 1 year ago. Possibly health state is perceived as better than 1 year ago, but still considerably worse compared to others. Nevertheless this rise in Health Change is in line with the reported improvements in other domains and these data highlight that symptomatic patients with FD/MAS may benefit from referral to a specialized tertiary center with multidisciplinary care.

New referrals generally reported worse scores for QoL and pain at baseline compared to chronic patients. Yet also in chronic patients clinically relevant improvements were observed, specifically in Role Physical and Emotional Wellbeing. Similarly in several other SF-36 domains and in pain interference smaller improvements were observed, which were neither significant nor clinically relevant, but we cannot rule out that a larger difference may be accomplished during a longer trajectory in the FD/MAS care pathway. The amelioration in chronic patients, although to a lesser extent, suggests that the improvements in new referrals could not merely be attributed to low baseline performance with large room for improvement or to regression to the mean, but confirms that the care pathway benefits QoL and pain, even in long-term care.

The COVID-19 pandemic might have influenced our results, as half of the patients (n = 49, 53%) completed the first questionnaire before and the second after the national social distancing measures in March 2020, whereas 22 patients (24%) completed both questionnaires before and 21 (23%) both during the pandemic. The major increase in Social Functioning, which we had not expected, may be explained by the fact that patients with low baseline scores for Social Functioning, mainly new referrals, experienced less difficulties in social life during the pandemic because of the absence of social gatherings.

A previous cross-sectional study on QoL and pain in FD/MAS has been conducted in our center by Majoor et al. [14] and showed better scores for QoL and pain compared to the present study. Accordingly our cohort demonstrated impairments in all domains of QoL in comparison to the general Dutch population, whereas Majoor et al. did not detect this for domains Role Emotional and Mental Health. Since QoL and pain are affected by FD subtype, skeletal burden, lesion location and age [14, 25, 38], variable study populations will result in a diversity of QoL and pain scores. Our study consisted of more patients with PFD and MAS, less with MFD and specified an extra subgroup of patients with CFD. Secondly, our study included new referrals, who may have a less extensive treatment history compared to patients under chronic care. Lastly, mainly patients with MFD reported impairments in our study compared to the previous study, which may all account for the difference between both studies.

Indeed of all FD subtypes in our cohort, patients with MFD demonstrated most impairments in domains Physical Function, Pain and Role Physical and most interference of pain with general activities. Both QoL and pain responded well to academic care and improved during follow-up. The largest reduction in pain interference with general activity was observed in patients with isolated craniofacial FD. On the contrary, patients with MAS experienced least improvement over time in both SF-36 and BPI scores. A hypothesis for the least severe FD subtypes benefiting most from therapy is that selection bias occurred when MFD or CFD patients with minor complaints were treated in non-academic centers and only those with severe complaints were referred and treated in academic centers, selecting the patients who are more likely to benefit from treatment. Nevertheless, this highlights that even patients with low skeletal burden may experience substantial negative FD-related consequences and may benefit from referral to a specialized center.

A secondary aim was to assess illness perceptions. The observed increase in QoL in our cohort could not be supported by a change in Illness Perceptions. The counselling, providing information and treatment in the LUMC care pathway surprisingly did not effectuate better perceived coherence of the disease or a higher perceived control over disease related complaints. The correlation between temporal change in SF-36 domain Role Physical and in pain interference is in line with the finding that QoL in our cohort was not influenced by altered illness perceptions but rather by improved pain management.

However QoL may even improve without change in pain scores. This was demonstrated by patients who had not received FD-related therapy during follow-up but still experienced a major improvement in Role Physical with an ES of 0.47 after 1 year, despite constant pain severity or interference. We hypothesize that the rise in QoL in our cohort may not only be explained by the effect of antiresorptive therapy or pain management but also by other factors, possibly multidisciplinary care, recognition of FD-related complaints, lifestyle advice or disease acceptance.

The COVID-19 pandemic could comprise a limitation of our study, as several negative consequences of the pandemic have been established in the general population including stress or worry [39], less physical activity [40], depressive symptoms, listlessness and decreased quality of life [41]. We were not able to assess the influence of several lockdowns on our study, but it might have exerted negative effects on Role Physical, Emotional Wellbeing, Energy/Fatigue, pain and pain interference. If so, this would result in an underestimation of the effect of the care pathway. Contrarily less physical activity could also benefit pain severity and interference. Another limitation is the discrepancy in sample sizes between referral groups, as the smaller sample of new referrals delivers results with more variance and more vulnerable to outliers. In addition the baseline scores of new referrals were used as proxy for the level of QoL and pain accomplished during secondary care before referral to our facility, but the true scores remain unknown. Finally our study comprised a heterogenous population of patients with FD/MAS with several subtypes, variable treatment history and various treatments started during follow-up. Ideally, to assess the added value of the care pathway, a randomized controlled trial should be conducted, where patients are randomized into treatment in the care pathway or in usual care, but limited resources hindered this design. Alternatively the approach in this study could be regarded as a strength, as it reflects the clinical heterogeneity present in every tertiary referral center and maintains external validity. This validity is further supported by the limited non-response bias, demonstrated by comparable characteristics of complete cases and non-responders. For these reasons results of this study may be generalized to patients with FD/MAS in other academic hospitals. Another strength of this study is the insight into questionnaires valuable for patients with FD/MAS, to screen for impairments not routinely addressed during standard medical consultations. Results were discussed with the Dutch Patient Association Fibrous Dysplasia to allow for improved and simplified questionnaire logistics in our FD/MAS care pathway. Lastly our study is the first to generate a longitudinal follow-up of PROMs in academic care and the first to combine outcomes such as QoL, mobility, pain and illness perceptions in patients with FD/MAS.

## Conclusion

We have established that patients with all subtypes of FD/MAS may suffer from negative consequences of their disease, and that the multidisciplinary coordinated care pathway for FD/MAS may improve QoL, pain and in particular pain interference, even though illness perceptions are unchanged. Patients who seem to benefit most from this care pathway are symptomatic new referrals without MAS comorbidities with low baseline performance. We recommend referral of patients with all subtypes of FD/MAS to tertiary academic centers and the implementation of the multidisciplinary care pathway for FD/MAS in those centers. Future studies should aim to determine the effect of the FD/MAS care pathway on time to diagnosis or therapy initiation, adherence to guidelines and uniformity of care. Lastly, attention is required for risk factors for an impaired QoL, for considerable pain interference, and for treatment-resistance in patients with FD/MAS.

## Supplementary Information

**Additional file 1**. Supplementary tables A–G.

**Additional file 2**. Figure A SF-36 at baseline and follow-up in patients with FD/MAS versus general Dutch population

**Additional file 3**. Figure B SF-36 domains at baseline and follow-up for FD-subtypes

**Additional file 4**. Figure C Pain scores in patients with moderate to severe pain over time across FD subtypes

**Additional file 5**. Figure D Pain scores in patients with pain score > 0 over time across FD subtypes

**Additional file 6**. Figure E SF-36 scores at baseline and follow-up across treatment during follow-up

**Additional file 7**. Figure F Pain scores at baseline and follow-up across treatment during follow-up

## Data Availability

The datasets generated and analysed during the current study are not publicly available due privacy legislation, but are available from the corresponding author on reasonable request.
