# Supplementary material for: A multidisciplinary care pathway improves quality of life and reduces pain in patients with fibrous dysplasia/McCune-Albright syndrome: a multicenter prospective observational study
Source: Orphanet J Rare Dis. 2022 Dec 17;17:439. doi: 10.1186/s13023-022-02588-z (PMC9758844; doi:10.1186/s13023-022-02588-z)
Supplement: Supplementary file 1 — Additional file 1. Supplementary tables A–G. [file 13023_2022_2588_MOESM1_ESM.docx]

**Additional file 1**

**Supplementary tables A – G**

| Table A: Quality of life scores of Short Form 36 * | | | | |
| --- | --- | --- | --- | --- |
|  | **Baseline**  mean (±SD) | **Follow up**  mean (±SD) | **Difference**  mean (95% CI) | **p-value** ^a^ |
| Physical function | | | | |
| Total (n=92) | 70.2 (24.3) | 74.3 (22.5) | + 4.2 (1.1-7.2) | p=0.008 |
| General population | 83.0 (22.8) |  |  |  |
| Difference mean (95% CI) | 12.8 (7.8-17.9) | 8.7 (4.0-13.3) |  |  |
| p-value ^b^  and effect size | p<0.001, ES 0.56 | p<0.001, ES 0.38 |  |  |
| Pain | | | | |
| Total (n=92) | 61.3 (26.1) | 64.9 (24.7) | + 3.5 (1.2-8.3) | p=0.142 |
| General population | 74.9 (23.4) |  |  |  |
| Difference mean (95% CI) | 13.6 (8.1-19.0) | 10.0 (4.9-15.1) |  |  |
| p-value ^b^ and effect size | p<0.001, ES 0.58 | p<0.001, ES 0.43 |  |  |
| Role limitations due to physical problems | | | | |
| Total (n=92) | 48.4 (44.4) | 62.5 (43.3) | + 14.1 (4.6-23.7) | p=0.004 |
| General population | 76.4 (36.3) |  |  |  |
| Difference mean (95% CI) | 28.0 (18.8-37.2) | 13.9 (4.9-22.9) |  |  |
| p-value ^b^  and effect size | p<0.001, ES 0.77 | p=0.03, ES 0.38 |  |  |
| Energy/fatigue | | | | |
| Total (n=92) | 50.9 (20.1) | 51.3 (17.4) | + 0.4 (- 4.1-5.0) | p=0.849 |
| General population | 68.6 (19.3) |  |  |  |
| Difference mean (95% CI) | 17.7 (13.6-21.9) | 17.3 (13.7-20.9) |  |  |
| p-value ^b^  and effect size | p<0.001, ES 0.92 | p<0.001, ES 0.90 |  |  |
| Emotional wellbeing | | | | |
| Total (n=92) | 54.7 (25.3) | 67.3 (16.0) | + 12.7 (7.3-18.0) | p<0.001 |
| General population | 76.8 (17.4) |  |  |  |
| Difference mean (95% CI) | 22.1 (16.9-27.4) | 9.5 (6.1-12.8) |  |  |
| p-value ^b^  and effect size | p<0.001, ES 1.27 | p<0.001, ES 0.55 |  |  |
| Role limitations due to emotional problems | | | | |
| Total (n=92) | 70.3 (38.7) | 77.2 (37.3) | + 6.9 (- 1.7-15.5) | p=0.115 |
| General population | 82.3 (32.9) |  |  |  |
| Difference mean (95% CI) | 12.0 (4.0-20.0) | 5.1 (- 2.6-12.8) |  |  |
| p-value ^b^  and effect size | p=0.004, ES 0.36 | p=0.191, ES 0.16 |  |  |
| Social Functioning | | | | |
| Total (n=92) | 71.3 (24.3) | 77.0 (21.6) | + 5.7 (1.1-10.3) | p=0.016 |
| General population | 84.0 (22.4) |  |  |  |
| Difference mean (95% CI) | 12.7 (7.6-17.7) | 7.0 (2.5-11.4) |  |  |
| p-value ^b^  and effect size | p<0.001, ES 0.57 | p=0.03, ES 0.31 |  |  |
| General Health | | | | |
| Total (n=92) | 55.8 (21.0) | 56.1 (19.6) | + 0.3 (- 2.6-3.2) | p=0.852 |
| General population | 70.7 (20.7) |  |  |  |
| Difference mean (95% CI) | 14.9 (10.5-19.2) | 14.6 (10.6-18.7) |  |  |
| p-value ^b^  and effect size | p<0.001, ES 0.72 | p<0.001, ES 0.71 |  |  |
| *: Higher scores reflect better QoL. Range: 0-100. a: Paired samples t-test, b: One-sample t-test, c: independent samples t-test | | | | |

| Table B: Quality of life scores of Short Form 36 across FD subtypes* | | | | |
| --- | --- | --- | --- | --- |
|  | **Baseline** mean (±SD) | **Follow up** mean (±SD) | **Difference** (95% CI) | **p-value** ^a^ |
| Physical function | | | | |
| MFD (n=22) | 63.2 (30.5) | 70.5 (26.7) | + 7.3 (- 1.1-15.6) | p=0.085 |
| CFD (n=16) | 84.4 (22.6) | 89.1 (14.3) | + 4.7 (- 1.2-10.6) | p=0.110 |
| PFD (n=37) | 69.9 (19.0) | 74.9 (16.5) | + 5.0 (0.7-9.2) | p=0.022 |
| MAS (n=17) | 66.5 (23.6) | 64.4 (28.4) | - 2.1 (- 9.8-5.7) | p=0.582 |
| p-value ^b^ | p=0.050 | p=0.011 |  |  |
| Pain | | | | |
| MFD (n=22) | 56.6 (30.7) | 61.8 (27.5) | + 5.2 (- 4.1-14.6) | p=0.262 |
| CFD (n=16) | 67.1 (24.2) | 75.1 (23.3) | + 8.0 (- 5.6-21.6) | p=0.227 |
| PFD (n=37) | 59.2 (26.2) | 63.0 (24.0) | + 3.8 (- 4.3-11.9) | p=0.345 |
| MAS (n=17) | 66.6 (21.2) | 63.3 (23.5) | + 3.4 (- 13.8-7.1) | p=0.505 |
| p-value ^b^ | p=0.493 | p=0.342 |  |  |
| Role limitations due to physical problems | | | | |
| MFD (n=22) | 40.9 (45.3) | 58.0 (42.5) | + 17.0 (- 4.1-38.2) | p=0.109 |
| CFD (n=16) | 48.4 (45.2) | 70.3 (40.0) | + 21.9 (1.9-41.9) | p=0.034 |
| PFD (n=37) | 50.0 (44.5) | 67.6 (43.2) | + 17.6 (4.0-31.2) | p=0.013 |
| MAS (n=17) | 54.4 (45.3) | 50.0 (47.6) | - 4.4 (- 35.0-26.2) | p=0.764 |
| p-value ^b^ | p=0.810 | p=0.448 |  |  |
| Energy/fatigue | | | | |
| MFD (n=22) | 53.2 (20.2) | 51.1 (19.8) | - 2.21 (- 12.7-8.6) | p=0.693 |
| CFD (n=16) | 55.6 (20.1) | 49.1 (16.8) | - 6.6 (- 18.0-4.9) | p=0.241 |
| PFD (n=37) | 46.5 (19.7) | 54.5 (16.4) | + 8.0 (1.8-14.2) | p=0.013 |
| MAS (n=17) | 52.9 (20.5) | 46.8 (17.0) | - 6.2 (- 18.0-5.6) | p=0.284 |
| p-value ^b^ | p=0.376 | p=0.445 |  |  |
| Emotional wellbeing | | | | |
| MFD (n=22) | 49.1 (28.5) | 63.3 (18.6) | + 14.2 (0.0-28.4) | p=0.050 |
| CFD (n=16) | 61.3 (22.9) | 64.5 (15.5) | + 3.3 (- 6.0-12.5) | p=0.464 |
| PFD (n=37) | 57.1 (22.3) | 69.3 (16.4) | + 12.2 (5.1-19.3) | p=0.001 |
| MAS (n=17) | 50.4 (29.3) | 70.8 (10.9) | + 20.5 (4.1-36.8) | p=0.017 |
| p-value ^b^ | p=0.401 | p=0.355 |  |  |
| Role limitations due to emotional problems | | | | |
| MFD (n=22) | 66.7 (38.5) | 66.7 (39.8) | 0.0 (- 21.9-21.9) | p=1.000 |
| CFD (n=16) | 66.7 (47.1) | 83.3 (32.2) | + 16.7 (- 8.5-41.8) | p=0.178 |
| PFD (n=37) | 70.3 (36.7) | 81.1 (37.3) | + 10.8 (- 0.9-22.5) | p=0.070 |
| MAS (n=17) | 78.4 (37.2) | 76.5 (38.7) | - 2.0 (- 21.6-17.6) | p=0.835 |
| p-value ^b^ | p=0.787 | p=0.464 |  |  |
| Social Functioning | | | | |
| MFD (n=22) | 65.3 (23.8) | 74.4 (23.0) | + 9.1 (0.0-18.2) | p=0.050 |
| CFD (n=16) | 66.4 (26.9) | 81.3 (20.4) | + 14.8 (2.9-26.8) | p=0.018 |
| PFD (n=37) | 74.0 (24.9) | 80.1 (21.3) | + 6.1 (- 1.6-13.7) | p=0.116 |
| MAS (n=17) | 77.9 (20.5) | 69.9 (21.2) | - 8.1 (- 17.4-1.3) | p=0.085 |
| p-value ^b^ | p=0.301 | p=0.321 |  |  |
| General Health | | | | |
| MFD (n=22) | 52.3 (26.9) | 52.3 (22.7) | 0 (- 6.0-6.0) | p=1.000 |
| CFD (n=16) | 53.8 (21.3) | 57.8 (17.7) | + 4.1 (- 3.1-11.2) | p=0.245 |
| PFD (n=37) | 59.7 (17.6) | 59.5 (18.2) | - 0.3 (- 4.7-4.2) | p=0.903 |
| MAS (n=17) | 53.8 (19.2) | 52.1 (19.9) | - 1.8 (- 10.4-6.9) | p=0.670 |
| p-value ^b^ | p=0.534 | p=0.433 |  |  |
| Health change compared to 1 year ago | | | | |
| MFD (n=22) | 40.9 (26.2) | 46.6 (22.2) | + 5.7 (- 9.2-20.6) | p=0.436 |
| CFD (n=16) | 46.9 (12.5) | 56.3 (21.4) | + 3.4 (- 3.4-22.1) | p=0.138 |
| PFD (n=37) | 46.6 (20.5) | 49.3 (19.1) | + 2.7 (- 6.4-11.9) | p=0.554 |
| MAS (n=17) | 47.1 (23.2) | 44.1 (20.8) | - 2.9 (- 20.5-14.6) | p=0.727 |
| p-value ^b^ | p=0.739 | p=0.360 |  |  |
| *: Higher scores reflect better QoL. Range: 0-100. a: Paired samples t-test, b: One way ANOVA | | | | |

|  | | | | |
| --- | --- | --- | --- | --- |
|  | | | | |
|  | | | | |
|  | | | | |
| Table C: Pain scores of BPI in patients with moderate-severe pain for FD-subtypes * | | | | |
|  | **Baseline**  median (Q1-Q3) | **Follow up**  median (Q1-Q3) | **Difference**  median (Q1-Q3) | **p-value ^a^** |
| Maximum pain | | | | |
| MFD (n=14) | 7.5 (4.75-8) | 6 (4-7.25) | - 0.5 (- 2.25-0.25) | p=0.101 |
| CFD (n=10) | 6.5 (4.75-7.25) | 5.5 (2-7) | - 1.5 (- 3-0.25) | p=0.048 |
| PFD (n=27) | 7 (4-8) | 7 (5-8) | 0 (- 2-2) | p=0.955 |
| MAS (n=11) | 7 (5-8) | 7 (6-8) | 0 (- 2-1) | p=0.764 |
| p-value  ^b^ | p=0.842 | p=0.442 |  |  |
| Average pain | | | | |
| MFD (n=14) | 5 (3.25-7) | 4.5 (2.75-7) | - 0.5 (- 2-0) | p=0.232 |
| CFD (n=10) | 3 (3-4.25) | 3 (2-4) | - 0.5 (- 1.5-1) | p=0.389 |
| PFD (n=27) | 4 (3-7) | 5 (2-6) | 0 (- 2-1) | p=0.145 |
| MAS (n=11) | 5 (3-6) | 4 (3-6) | 0 (- 2-1) | p=0.256 |
| p-value  ^b^ | p=0.165 | p=0.175 |  |  |
| Interference of pain with general activity | | | | |
| MFD (n=14) | 7 (4-8) | 6 (1.75-8) | - 0.5 (- 3.25-1.25) | p=0.227 |
| CFD (n=10) | 4 (1.75-7.25) | 1 (0-2) | - 2 (- 5.25—0.75) | p=0.012 |
| PFD (n=27) | 5 (1-6) | 4 (0-6) | 0 (- 2-1) | p=0.357 |
| MAS (n=11) | 5 (2-8) | 5 (3-7) | 1 (- 2-2) | p=1.00 |
| p-value  ^b^ | p=0.138 | p=0.017 |  |  |
| *: in patients with maximum or average pain ≥4. Higher scores=more pain and more interference. Range 0-10  a: Wilcoxon signed rank test, comparison between time points, b: Kruskal Wallis test   \| Table D: Pain scores of BPI in patients with pain score >0 for FD subtypes * \| \| \| \| \| \| \| --- \| --- \| --- \| --- \| --- \| --- \| \|  \| **Baseline**  median (Q1-Q3) \| **Follow up**  median (Q1-Q3) \| **Difference**  median (Q1-Q3) \| **p-value** ^a^ \| \| \| Maximum pain \| \| \| \| \| \| \| MFD (n=21) \| 5 (2.5-8) \| 5 (2.5-7) \| -1 (- 2-1) \| p=0.364 \| \| \| CFD (n=14) \| 5.5 (2-7) \| 5 (1.75-6.25) \| -0.5 (- 2.25-1) \| p=0.323 \| \| \| PFD (n=33) \| 7 (3-7.5) \| 6 (3.5-8) \| 0 (- 1-1.5) \| p=0.782 \| \| \| MAS (n=16) \| 5 (1.25-7.75) \| 6 (3.25-7) \| 0 (- 1-2) \| p=0.438 \| \| \| p-value  ^b^ \| p=0.841 \| p=0.405 \|  \|  \| \| \| Average pain \| \| \| \| \| \| \| MFD (n=21) \| 4 (1.5-6.5) \| 4 (2-6.5) \| 0 (- 1-0) \| p=0.906 \| \| \| CFD (n=14) \| 3 (2-4) \| 2.5 (1.75-3.25) \| -0.5 (- 1-1) \| p=0.330 \| \| \| PFD (n=33) \| 4 (3-6) \| 4 (2-6) \| 0 (- 1-1) \| p=0.371 \| \| \| MAS (n=16) \| 3.5 (1.25-5.75) \| 3 (2-4.75) \| 0 (- 1.5-1) \| p=0.569 \| \| \| p-value  ^b^ \| p=0.460 \| p=0.184 \|  \|  \| \| \| Interference of pain with general activity \| \| \| \| \| \| \| MFD (n=21) \| 4 (0-7) \| 5 (0-6.5) \| 0 (- 2-2) \| p=0.825 \| \| \| CFD (n=14) \| 2.5 (0-5.5) \| 1 (0-2) \| -1 (- 4.25-0) \| p=0.036 \| \| \| PFD (n=33) \| 4 (1-6) \| 3 (1-6) \| 0 (- 2-1) \| p=0.232 \| \| \| MAS (n=16) \| 2.5 (1.25-6.75) \| 3 (2-5.75) \| 1 (- 1.75-1.75) \| p=0.863 \| \| \| p-value  ^b^ \| p=0.768 \| P=0.042 \|  \|  \| \| \| *: in patients with pain at baseline, maximum or average pain >0. Higher scores=more pain and more interference. Range 0-10  a: Wilcoxon signed rank test, comparison between time points, b: Mann Whitney U test, comparison between hospitals  c: Kruskal Wallis test, comparison between diagnosis types   \| Table E: Change in SF-36 scores for treatment groups during follow-up * \| \| \| \| \| \| --- \| --- \| --- \| --- \| --- \| \|  \| **Baseline**  mean (±SD) \| **Follow up**  mean (±SD) \| **Difference**  (95% CI) \| **p-value** ^a^ \| \| SF-36 physical function \| \| \| \| \| \| FD-related therapy (n=50) \| 64.7 (24.1) \| 70.7 (23.8) \| + 6.0 (1-7-10.3) \| p=0.007 \| \| No therapy (n=42) \| 76.7 (23.2) \| 78.7 (20.4) \| + 2.0 (- 2.3-6.4) \| p=0.355 \| \| p-value ^b^ \| p=0.018 \| p=0.091 \|  \|  \| \| SF-36 role physical \| \| \| \| \| \| FD-related therapy (n=50) \| 44.0 (44.8) \| 52.5 (45.2) \| + 8.5 (- 5.0-22.0) \| p=0.212 \| \| No therapy (n=42) \| 53.6 (44.0) \| 74.4 (38.1) \| + 20.8 (7.2-34.5) \| p=0.004 \| \| p-value ^b^ \| p=0.306 \| p=0.013 \|  \|  \| \| Emotional wellbeing \| \| \| \| \| \| FD-related therapy (n=50) \| 50.6 (26.5) \| 69.0 (17.4) \| + 18.5 (10.5-26.4) \| p<0.001 \| \| No therapy (n=42) \| 59.5 (23.3) \| 65.2 (14.1) \| + 5.7 (- 1.0-12.4) \| p=0.092 \| \| p-value ^b^ \| p=0.091 \| p=0.259 \|  \|  \| \| Social functioning \| \| \| \| \| \| FD-related therapy (n=50) \| 68.5 (24.1) \| 75.8 (21.6) \| + 7.3 (0.6-13.9) \| p=0.033 \| \| No therapy (n=42) \| 74.7 (24.5) \| 78.6 (21.8) \| + 3.9 (-2.8-10.5) \| p=0.246 \| \| p-value ^b^ \| p=0.225 \| p=0.536 \|  \|  \| \| *: FD-related therapy: antiresorptive therapy or surgery  a: paired samples t-test, parametric comparison between time points  b: independent samples t-test, parametric comparison between groups \| \| \| \| \| \| \| \| \| \| | | | | |

| Table F: Change in pain scores for treatment groups during follow-up * | | | | |
| --- | --- | --- | --- | --- |
|  | **Baseline**  median (Q1-Q3) | **Follow up**  median (Q1-Q3) | **Difference**  median (Q1-Q3) | **p-value** ^a^ |
| Maximum pain | | | | |
| FD-related therapy (n=46) | 7 (4-8) | 6 (4-8) | 0 (- 2-1) | p=0.587 |
| No therapy (n=38) | 4 (2-7) | 5 (2-7) | 0 (- 2-2) | p=0.967 |
| p-value ^b^ | p=0.021 | p=0.035 |  |  |
| Average pain | | | | |
| FD-related therapy (n=46) | 4 (3-6) | 3 (2-6) | 0 (- 2-1) | p=0.089 |
| No therapy (n=38) | 3 (1-4.25) | 3 (1-5) | 0 (- 1-1) | p=0.912 |
| p-value ^b^ | p=0.014 | p=0.0147 |  |  |
| Pain interference | | | | |
| FD-related therapy (n=46) | 5 (2-7) | 3 (1-6.25) | 0 (- 3-1) | p=0.061 |
| No therapy (n=38) | 2 (0-4.25) | 2 (0-5) | 0 (- 1-1) | p=0.686 |
| p-value ^b^ | p=0.002 | p=0.063 |  |  |
| *: FD-related therapy: antiresorptive therapy or surgery, in patients with pain score >0  a: Wilcoxon signed rank test, non-parametric comparison between time points  b: Mann Whitney U test, non-parametric comparison between groups | | | | |

| Table G: Illness perceptions * | | | |
| --- | --- | --- | --- |
|  | **Baseline** mean (±SD) | **Follow up** mean (±SD) | **p-value** ^a^ |
| Timeline acute/chronic | | | |
| Total (n=86) | 18.7 (1.8) | 18.8 (1.6) | p=0.574 |
| New (n=25) | 18.6 (1.7) | 18.5 (2.1) | p=0.937 |
| Chronic (n=61) | 18.7 (1.9) | 18.9 (1.4) | p=0.455 |
| p-value ^b^ | p=0.766 | p=0.323 |  |
| MFD (n=20) | 18.8 (2.0) | 18.8 (1.7) | p=1.000 |
| CFD (n=15) | 18.8 (1.4) | 19.3 (1.2) | p=0.192 |
| PFD (n=35) | 18.5 (2.1) | 18.7 (1.8) | p=0.811 |
| MAS (n=16) | 18.6 (1.2) | 18.6 (1.5)0 | p=1.000 |
| p-value ^c^ | p=0.963 | p=0.557 |  |
| Timeline cyclical | | | |
| Total (n=86) | 12.1 (3.9) | 12.3 (4.1) | p=0.629 |
| New (n=25) | 11.2 (3.3) | 11.4 (4.3) | p=0.839 |
| Chronic (n=61) | 12.5 (4.1) | 12.7 (3.9) | p=0.656 |
| p-value ^b^ | p=0.179 | p=0.183 |  |
| MFD (n=20) | 11.5 (3.5) | 10.5 (4.3) | p=0.311 |
| CFD (n=15) | 10.6 (3.9) | 11.2 (4.5) | p=0.580 |
| PFD (n=35) | 12.6 (3.9) | 13.2 (3.6) | p=0.279 |
| MAS (n=16) | 13.3 (4.3) | 13.7 (3.6) | p=0.562 |
| p-value ^c^ | p=0.189 | p=0.032 |  |
| Consequences | | | |
| Total (n=86) | 15.4 (3.5) | 14.9 (3.6) | p=0.047 |
| New (n=25) | 15.3 (3.3) | 14.4 (3.1) | p=0.114 |
| Chronic (n=61) | 15.5 (3.6) | 15.1 (3.7) | p=0.205 |
| p-value ^b^ | p=0.830 | p=0.439 |  |
| MFD (n=20) | 15.0 (3.5) | 14.1 (3.4) | p=0.036 |
| CFD (n=15) | 13.8 (2.5) | 13.4 (2.3) | p=0.546 |
| PFD (n=35) | 15.5 (3.1) | 15.2 (3.3) | p=0.521 |
| MAS (n=16) | 17.3 (4.3) | 16.8 (4.5) | p=0.333 |
| p-value ^c^ | p=0.043 | p=0.035 |  |
| Personal control | | | |
| Total (n=86) | 16.7 (2.5) | 16.5 (2.2) | p=0.542 |
| New (n=25) | 16.2 (2.7) | 16.2 (2.2) | p=1.000 |
| Chronic (n=61) | 16.9 (2.4) | 16.6 (2.2) | p=0.427 |
| p-value ^b^ | p=0.268 | p=0.412 |  |
| MFD (n=20) | 17.7 (2.1) | 17.4 (2.1) | p=0.635 |
| CFD (n=15) | 15.2 (2.9) | 15.5 (2.2) | p=0.691 |
| PFD (n=35) | 16.7 (2.3) | 16.4 (1.6) | p=0.487 |
| MAS (n=16) | 16.8 (2.5) | 16.6 (2.9) | p=0.835 |
| p-value ^c^ | p=0.034 | p=0.066 |  |
| Treatment control | | | |
| Total (n=86) | 15.4 (2.3) | 15.8 (2.2) | p=0.204 |
| New (n=25) | 15.2 (2.6) | 15.6 (2.4) | p=0.507 |
| Chronic (n=61) | 15.5 (2.2) | 15.9 (2.1) | p=0.281 |
| p-value ^b^ | p=0.583 | p=0.636 |  |
| MFD (n=20) | 15.7 (1.6) | 15.9 (1.7) | p=0.778 |
| CFD (n=15) | 15.5 (1.8) | 15.9 (2.8) | p=0.565 |
| PFD (n=35) | 15.0 (2.4) | 15.7 (2.4) | p=0.113 |
| MAS (n=16) | 16.0 (3.3) | 16.0 (1.7) | p=1.000 |
| p-value ^c^ | p=0.472 | p=0.952 |  |
| Illness coherence | | | |
| Total (n=86) | 14.0 (2.0) | 14.1 (1.8) | p=0.614 |
| New (n=25) | 13.8 (1.9) | 14.6 (2.0) | p=0.142 |
| Chronic (n=61) | 14.1 (2.0) | 13.9 (1.8) | p=0.608 |
| p-value ^b^ | p=0.639 | p=0.156 |  |
| MFD (n=20) | 14.3 (2.0) | 14.4 (1.5) | p=0.748 |
| CFD (n=15) | 13.4 (1.8) | 14.4 (2.5) | p=0.221 |
| PFD (n=35) | 14.1 (2.2) | 13.9 (1.7) | p=0.746 |
| MAS (n=16) | 14.1 (1.9) | 13.9 (1.9) | p=0.714 |
| p-value ^c^ | p=0.615 | p=0.712 |  |
| Emotional response | | | |
| Total (n=86) | 13.4 (3.5) | 12.6 (4.1) | p=0.042 |
| New (n=25) | 14.0 (3.6) | 13.7 (4.6) | p=0.726 |
| Chronic (n=61) | 13.2 (3.4) | 12.2 (3.9) | p=0.024 |
| p-value ^b^ | p=0.346 | p=0.130 |  |
| MFD (n=20) | 14.5 (3.9) | 13.3 (3.9) | p=0.091 |
| CFD (n=15) | 14.0 (3.3) | 11.1 (3.7) | p=0.005 |
| PFD (n=35) | 13.2 (3.5) | 13.0 (4.3) | p=0.711 |
| MAS (n=16) | 12.0 (2.9) | 12.6 (4.3) | p=0.488 |
| p-value ^c^ | p=0.171 | p=0.378 |  |
| * Range of scores: 6-30 for timeline acute/chronic, consequences, personal control and emotional representation, 4-20 for timeline cyclical, 5-25 for treatment control and illness coherence. High scores for personal/treatment control and illness coherence indicate positive beliefs about controllability and understanding of the disease. High scores on timeline acute/chronic/cyclical, consequences and emotional response represent strong beliefs on the acute/chronic/cyclical nature and on negative consequences and emotions caused by the disease. a: paired samples t-test; comparison between time points, b: independent samples t-test; comparison between hospitals, c: one way ANOVA; comparison between diagnosis types | | | |

| Table H. Missing data analysis  Comparison of characteristics of all patients included > 1 year in PROFID study | | | | |
| --- | --- | --- | --- | --- |
|  | **Completers of both surveys**  N=92 | **Dropout after 1st survey**  N=26 | **No surveys completed**  N=6 | **p-value** |
| Age at baseline (mean, ±SD) | 44.8 (15.2) | 34.9 (15.8) | 41.5 (19.4) | 0.018 ^a^ |
| Sex, female (number, %) | 61 (66.3%) | 20 (76.9%) | 3 (50%) | 0.344 ^b^ |
| Type of FD (number, %)  MFD non CFD  Isolated CFD  PFD  MAS | 22 (23.9%)  16 (17.4%)  37 (40.2%)  17 (18.5%) | 6 (23.1%)  7 (26.9%)  7 (26.9%)  6 (23.1%) | 1 (16.7%)  2 (33.3%)  3 (50%)  0 | 0.657 ^b^ |
| Skeletal Burden Score (median, Q1-Q3) | 3.2 (1.2-16.9) | 2.8 (1.3-17.7) | 7.8 (2.5-18.8) | 0.806 ^c^ |
| Baseline QoL (mean, SD)  Physical function  Vitality  General Health | 70.2 (24.3)  50.9 (20.1)  55.8 (21.0) | 69.6 (26.5)  48.8 (18.9)  52.5 (21.0) | N/A | 0.921 ^d^  0.647 ^d^  0.478 ^d^ |
| Baseline average pain score (median, Q1-Q3) | 3 (2-5) | 4 (2-5) | N/A | 0.824 ^d^ |
| a: one-way ANOVA, b: Chi-square test, c: Kruskall Wallis test, d: Mann-Whitney U test | | | | |
