# Supplementary figures and images for: A multidisciplinary care pathway improves quality of life and reduces pain in patients with fibrous dysplasia/McCune-Albright syndrome: a multicenter prospective observational study
Source: Orphanet J Rare Dis. 2022 Dec 17;17:439. doi: 10.1186/s13023-022-02588-z (PMC9758844; doi:10.1186/s13023-022-02588-z)

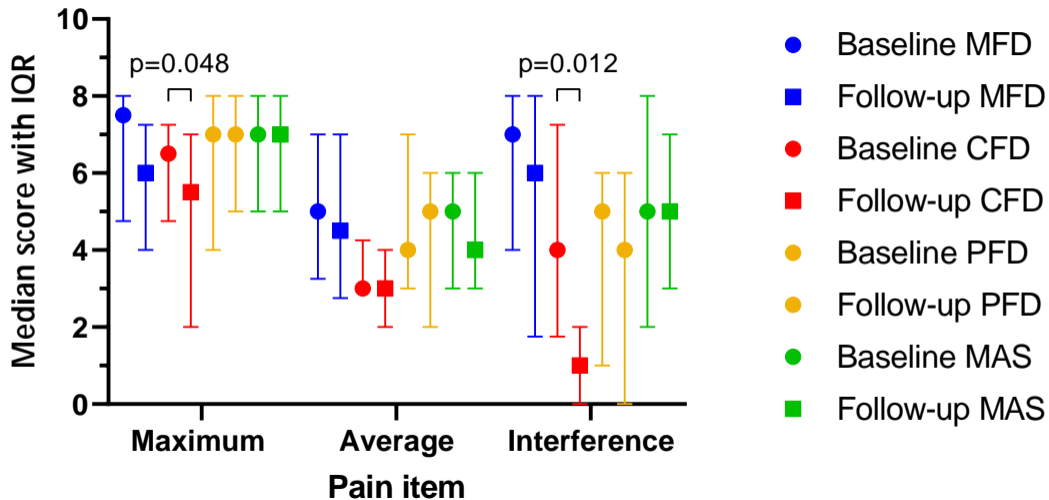

Supplement: Supplementary file 4 — Additional file 4. Figure C Pain scores in patients with moderate to severe pain over time across FD subtypes [file 13023_2022_2588_MOESM4_ESM.pdf]

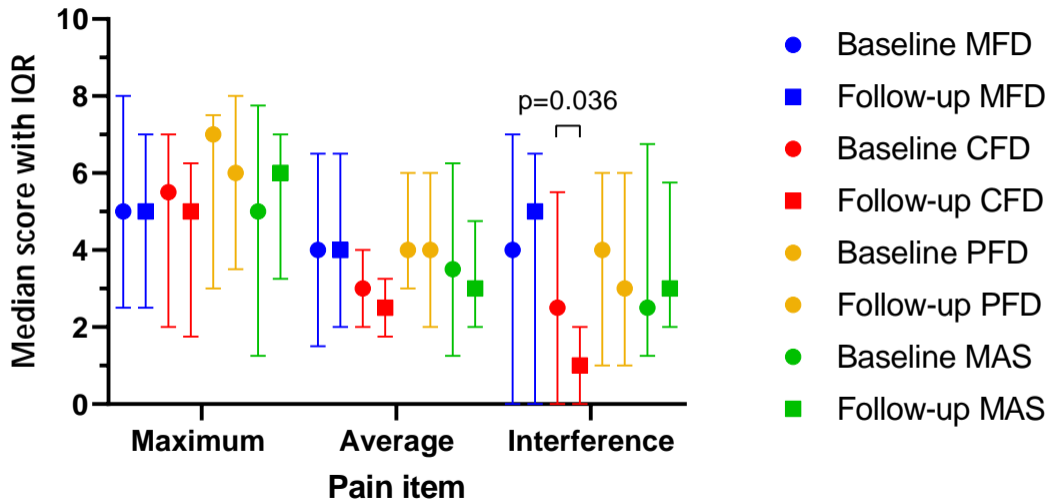

Supplement: Supplementary file 5 — Additional file 5. Figure D Pain scores in patients with pain score > 0 over time across FD subtypes [file 13023_2022_2588_MOESM5_ESM.pdf]

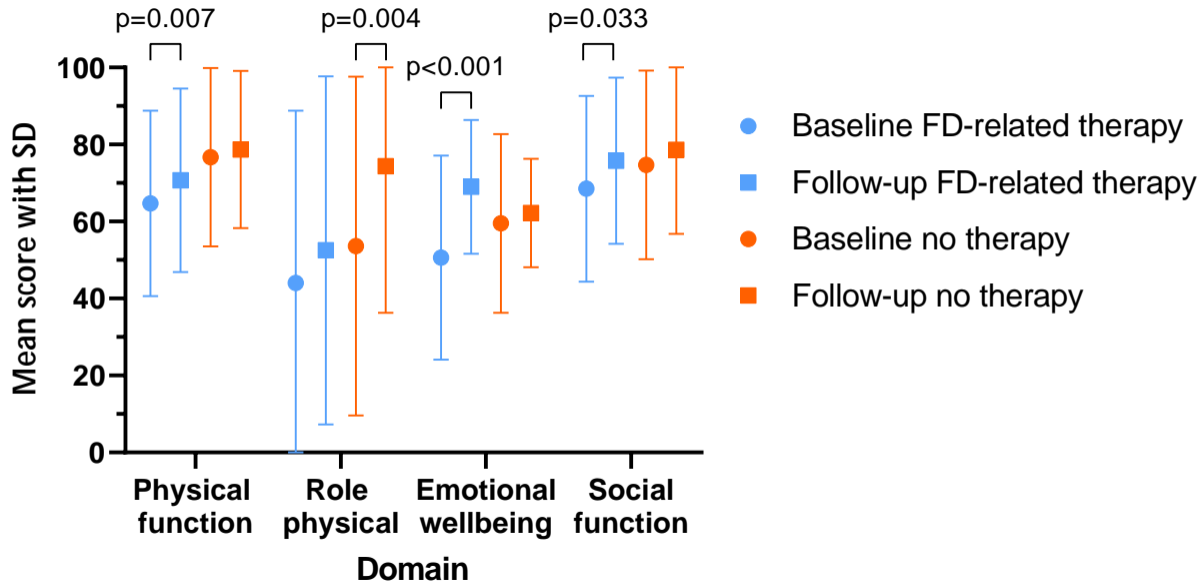

Supplement: Supplementary file 6 — Additional file 6. Figure E SF-36 scores at baseline and follow-up across treatment during follow-up [file 13023_2022_2588_MOESM6_ESM.pdf]

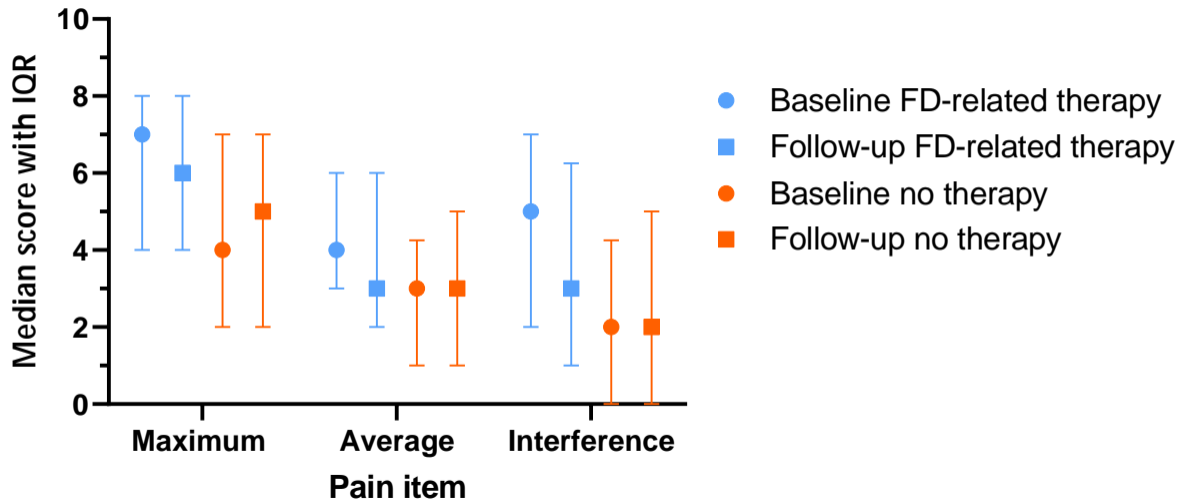

Supplement: Supplementary file 7 — Additional file 7. Figure F Pain scores at baseline and follow-up across treatment during follow-up [file 13023_2022_2588_MOESM7_ESM.pdf]
