# Supplementary material for: A multidisciplinary care pathway improves quality of life and reduces pain in patients with fibrous dysplasia/McCune-Albright syndrome: a multicenter prospective observational study
Source: Orphanet J Rare Dis. 2022 Dec 17;17:439. doi: 10.1186/s13023-022-02588-z (PMC9758844; doi:10.1186/s13023-022-02588-z)
Supplement: Supplementary file 3 [file 13023_2022_2588_MOESM2_ESM.docx]

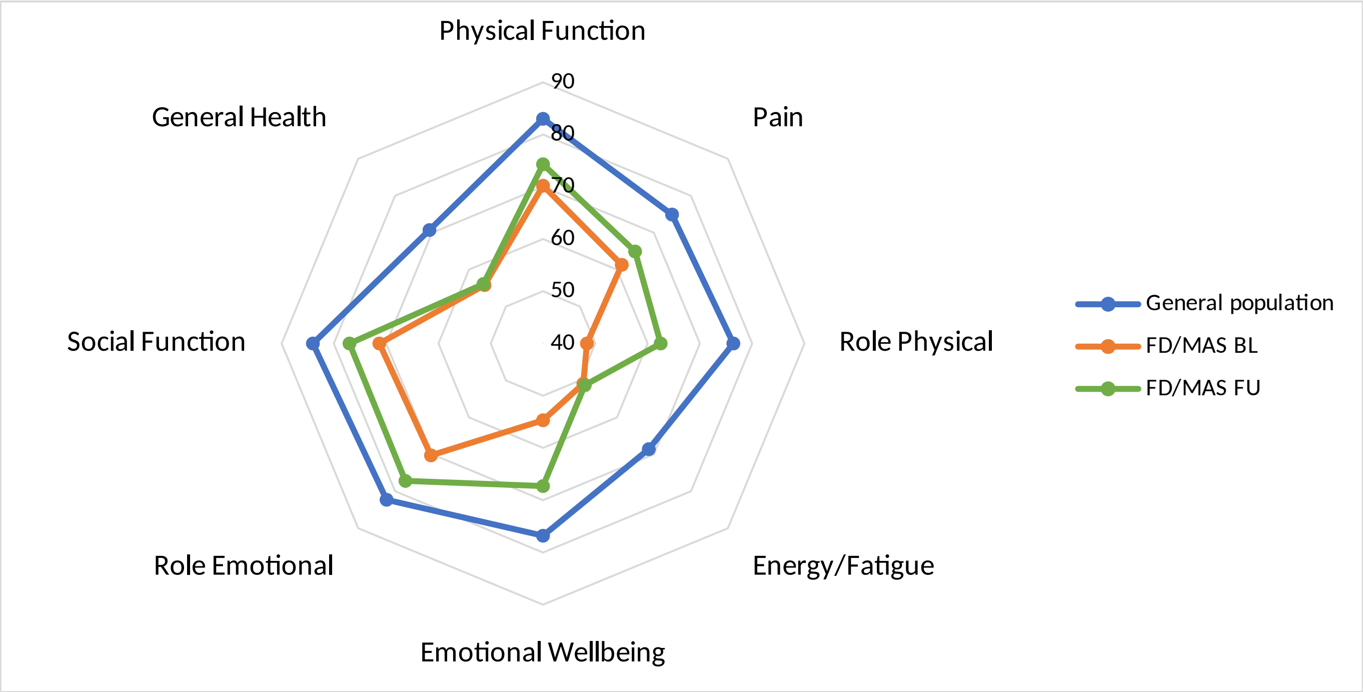

Physical Function
Pain
Role Physical
Energy/Fatigue
Emotional Wellbeing
Role Emotional
Social Function
General Health
90
80
70
60
50
40
General population
FD/MAS BL
FD/MAS FU
