# Supplementary material for: A multidisciplinary care pathway improves quality of life and reduces pain in patients with fibrous dysplasia/McCune-Albright syndrome: a multicenter prospective observational study
Source: Orphanet J Rare Dis. 2022 Dec 17;17:439. doi: 10.1186/s13023-022-02588-z (PMC9758844; doi:10.1186/s13023-022-02588-z)
Supplement: Supplementary file 4 [file 13023_2022_2588_MOESM3_ESM.docx]

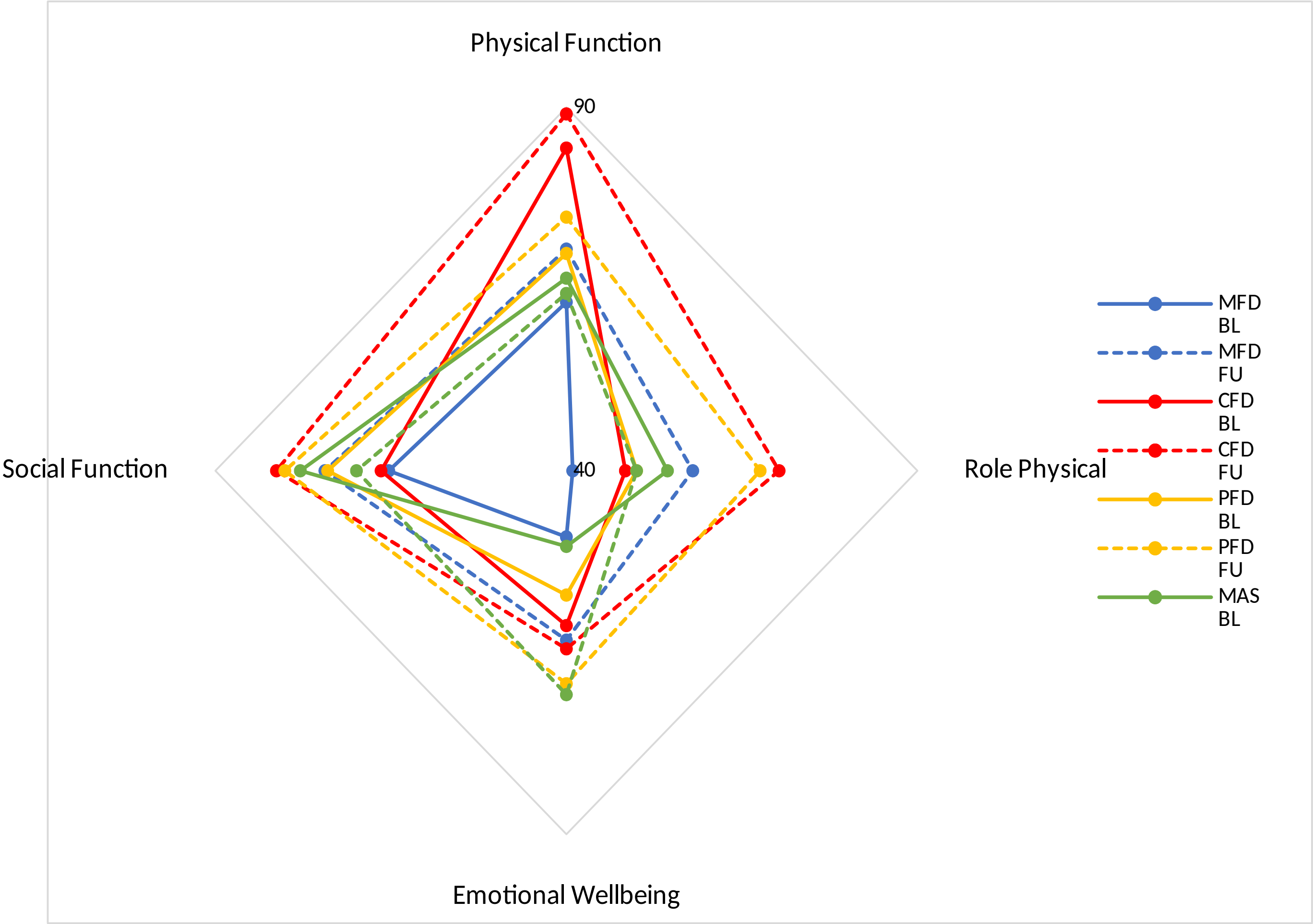

Physical Function
90
40
Role Physical
Emotional Wellbeing
Social Function
MFD BL
MFD FU
CFD BL
CFD FU
PFD BL
PFD FU
MAS BL
